# Suspected cholinergic toxicity due to cevimeline hydrochloride and *Bacopa monnieri* interaction: a case report

**DOI:** 10.1186/s13256-022-03479-4

**Published:** 2022-06-29

**Authors:** Blake Acquarulo, Priya Tandon, Carolyn M. Macica

**Affiliations:** Frank H. Netter MD School of Medicine at Quinnipiac University, North Haven, CT USA

**Keywords:** Sjögren’s, Herbal supplement, Cevimeline, Acetylcholine, Drug interaction, Adverse effects

## Abstract

**Background:**

Muscarinic agonists are indicated for the treatment of many conditions including ileus, urinary retention, glaucoma, and Sjögren’s syndrome. Due to their lack of tissue specificity, these drugs can lead to undesirable side effects at off-target sites and may be potentiated by supplements that impact the half-life of these drugs.

**Case presentation:**

A 58-year-old Caucasian female with history of Sjögren’s syndrome, who was being managed with cevimeline, presented to the primary care office with reported hyperhidrosis, malaise, nausea, and tachycardia. She reported taking an herbal supplement containing *B. monnieri* and phosphatidylserine the previous night. It has been previously demonstrated that *B. monnieri* alters cytochrome P450 enzymes. Electrocardiogram showed no acute ST–T changes. Clinical improvement occurred with hydration and discontinuation of the supplement.

**Conclusions:**

To our knowledge, there has only been one other documented cevimeline overdose, and it was not associated with an herbal supplementation interaction. Physicians should actively elicit herbal supplement information from patients to anticipate possible drug–herb interactions. An additional consideration of clinical relevance is the known genetic variability that may affect drug responsiveness due to differences in metabolism and half-life of drugs that arise from common genetic variants of cytochrome P450 genes.

## Background

Sjögren’s syndrome (SjS) is a long-term rheumatological autoimmune disorder that has both nonglandular and glandular symptoms. This includes eye and mouth dryness due to mononuclear cell infiltration of the exocrine glands, particularly the lacrimal and salivary glands, which can result in long-term ocular and oral manifestations [1]. SjS most often manifests between the ages of 45 and 55 years, disproportionately affecting females at a 9:1 ratio, and is estimated to affect 0.5–1.0% of the population [2]. The etiology of SjS is unknown, but several factors, including detection of Epstein–Barr virus in salivary glands, has been implicated in the autoimmune response [3]. A genetic predisposition to the development of SjS has been established, but no specific inheritance pattern has been identified. Like other complex autoimmune disorders, evidence suggests multifactorial origins including pathogenic variants or epigenetic modifiers of class I and more significantly class II human leukocyte antigens (HLAs), displaying ethnic heterogeneity [4]. SjS susceptibility has also correlated with genes that regulate the innate and adaptive immune systems and may contribute to immune response dysregulation [4].

Cevimeline hydrochloride is a cholinergic agonist that increases glandular secretion and is indicated in the treatment of xerostomia in patients with SjS [5]. The drug acts by binding and activating M_3_ receptors, resulting in increased secretions from exocrine glands, including the salivary and sweat glands [5]. However, muscarinic receptor drug precision is problematic because of broad receptor tissue distribution, giving rise to off-target adverse effects. In addition, genetic variation of the enzymes that metabolize drugs and drug–drug interactions may strongly influence drug half-life.

Alternative plant-derived herbal medications have been used for medicinal purposes for centuries, and the current worldwide annual market for herbal products is approximately US $60 billion [6]. We report a patient who took the recommended adult dosage of a supplement promoted to enhance brain function with active ingredients listed as *Bacopa monnieri* and phosphatidylserine. *B. monnieri*, also known as brahmi, is an herb often used in traditional Asian Indian Ayurvedic medicine to improve cognitive enhancement [7]. *B. monnieri* has been studied as an acetylcholinesterase inhibitor in animal and *in vitro* models [7]. Similarly, phosphatidylserine is also marketed to enhance memory and cognitive function in the elderly, and animal studies indicate phosphatidylserine potentiates acetylcholine levels in the central nervous system (CNS) [8].

In this report, we describe the case of a patient with Sjögren's syndrome who had a suspected cevimeline overdose with cholinergic toxicity after consuming an herbal supplement containing *B. monnieri* and phosphatidylserine. To our knowledge, there has only been one other documented cevimeline overdose, and that case was not associated with herbal supplementation interaction [9].

## Case presentation

A 58-year-old Caucasian female with a prior diagnosis of SjS and subacute cutaneous lupus erythematosus presented to our primary care office, where she is an established patient. The patient was prior prescribed cevimeline 30 mg three times a day since diagnosis. She was additionally prescribed 150 mg daily dose of hydroxychloroquine for management of lupus. The patient’s personal medical history also included a tear duct blockage 5 years prior and family history of rheumatoid arthritis.

On presentation to the clinic, the patient reported a 1-day complaint of “heart racing, sweating, unease, headache, dizziness, nausea” and the “physical feeling of wanting to have a bowel movement” that continued for several hours. She reported that, the previous night, she had taken her usual medication of 30 mg of cevimeline, but included two gummy formulation tablets of the *B. monnieri* and phosphatidylserine-containing herbal supplement. She did not consult with primary care regarding the herbal supplement. Within 30 minutes of ingestion, the patient reported experiencing acute onset of tachycardia, hyperhidrosis, malaise, nausea, and tenesmus. She decided to “sleep off” her symptoms and called our office the next morning when the malaise and nausea had not fully resolved.

A physical examination showed a blood pressure of 127/77 and a pulse oximetry reading at room air of 94%, whereas all other parameters were within normal range. She was fully alert and oriented without any neurological deficits. Pupils were equal, round, and reactive to light and accommodation. On auscultation, patient had regular heart rate and rhythm, normal S1 and S2 heart sounds without murmur, gallop, or rub. Lung and abdominal examination were unremarkable.

An electrocardiogram (ECG) was performed and showed no acute ST–T changes and normal heart rate of 83. The patient was advised to hydrate and to discontinue the herbal supplement. On telephone follow-up the next day, the patient reported that her symptoms had subsided and have since fully resolved. Resolution of symptoms was consistent with the clearance of the drug following a single dose.

## Discussion

We report the case of a potential drug–herb interaction involving cevimeline hydrochloride and *Bacopa monnieri* that resulted in acute suspected cholinergic toxicity. Currently there are several listed drug–herb interactions between cevimeline hydrochloride and various herbal supplements. To our knowledge, *B. monnieri* and its potential drug–herb interaction with cevimeline has not previously been demonstrated in literature. It has been previously demonstrated that *B. monnieri* alters cytochrome P450 (CYP) enzymes. A study of the effect of *B. monnieri* on the five major CYP isoforms *in vitro* demonstrated that *B. monnieri* acts as a competitive inhibitor of CYP3A4 and a weak inhibitor of CYP2D6 [10], both of which are responsible for metabolizing a majority of drugs in humans. Cevimeline is metabolized primarily by the hepatic isozymes CYP2D6, CYP3A3, and CYP3A4 [11].

Therefore, we hypothesize that our patient’s symptomatology is consistent with a potential cholinergic toxicity due to the effect of *B. monnieri* extract on CYP isoforms that metabolize cevimeline, resulting in elevated therapeutic concentrations of plasma cevimeline. An additional consideration of clinical relevance is the known genetic variability that may affect drug responsiveness due to differences in metabolism of drugs that arise from common genetic variants of CYP genes. While genetic testing is required for prescription drugs with a narrow therapeutic index to minimize potential toxicities and to ensure efficacy due to CYP variants, genetic variability is currently often unknown for most individuals and may impact the therapeutic window of both prescription drugs and supplements.

Currently, the Food and Drug Administration (FDA) categorizes herbal supplements with vitamins and minerals as “dietary supplements,” therefore they are regulated differently than pharmaceuticals [12]. The FDA only monitors for safety and adverse events after dietary supplements are introduced to the market [12]. Therefore, herbal supplements can be produced and sold without having to first demonstrate their safety and efficacy profiles to the FDA [12]. Herbal supplements may be viewed by patients as “natural” and therefore without harm to the patient [13]. Of note, patients may not disclose use of herbal supplements to their clinicians. Indeed, a previous study identified multiple demographic variables that impact nondisclosure. Although they reported that individuals with chronic medical conditions who access healthcare more frequently and who are managed with prescription drugs have a higher likelihood of disclosure, the rate is still less than 50% [14]. Importantly, improved communication between providers and their patients regarding herbal medication usage could help anticipate possible drug–herb interactions [15]. This also highlights the importance of careful patient history-taking by healthcare providers.

A limitation of our case report is the lack of detailed information on the composition of the supplement taken by the patient and suspected to have caused the described event. Additionally, it is not possible to establish a definitive causal link regarding the onset of the patient’s symptoms and the consumption of a supplement containing *B. monnieri*. However, even in the absence of knowing the patient’s CYP metabolic profile (not standard), we can hypothesize that, in this female patient who had previously been taking cevimeline hydrochloride for years with no adverse events, administration of a supplement containing *B. monnieri* may have resulted in cholinergic toxicity and the patient’s resulting symptomatology.

## Conclusions

To our knowledge, this is the first documented case of a suspected cevimeline overdose associated with herbal supplementation. Physicians should actively elicit herbal supplement information from patients and continue to stay up to date on the literature regarding potential drug–herb interaction, especially among those taking medications for chronic illnesses.

## Data Availability

Not applicable.

## Abbreviations

SjS: Sjögren’s syndrome
CNS: Central nervous system
ECG: Electrocardiogram
CYP: Cytochrome
FDA: Food and Drug Administration
